# Improving Challenge/Skill Ratio in a Multimodal Interface by Simultaneously Adapting Game Difficulty and Haptic Assistance through Psychophysiological and Performance Feedback

**DOI:** 10.3389/fnins.2017.00242

**Published:** 2017-05-01

**Authors:** Carlos Rodriguez-Guerrero, Kristel Knaepen, Juan C. Fraile-Marinero, Javier Perez-Turiel, Valentin Gonzalez-de-Garibay, Dirk Lefeber

**Affiliations:** ^1^Robotics and Multibody Mechanics, Flanders Make, Vrije Universiteit BrusselBrussels, Belgium; ^2^Institute for Movement and Neurosciences, German Sport University CologneCologne, Germany; ^3^Human Physiology Research Group, Vrije Universiteit BrusselBrussels, Belgium; ^4^Biomedical Engineering, Fundacion CARTIF, Centro Tecnologico de BoecilloValladolid, Spain; ^5^Department of Statistics and Operative Research, Universidad de ValladolidValladolid, Spain

**Keywords:** psychophysiology, human–robot interaction, biocooperative, rehabilitation robotics, haptics, multimodal interfaces, biomechatronics

## Abstract

In order to harmonize robotic devices with human beings, the robots should be able to perceive important psychosomatic impact triggered by emotional states such as frustration or boredom. This paper presents a new type of biocooperative control architecture, which acts toward improving the challenge/skill relation perceived by the user when interacting with a robotic multimodal interface in a cooperative scenario. In the first part of the paper, open-loop experiments revealed which physiological signals were optimal for inclusion in the feedback loop. These were heart rate, skin conductance level, and skin conductance response frequency. In the second part of the paper, the proposed controller, consisting of a biocooperative architecture with two degrees of freedom, simultaneously modulating game difficulty and haptic assistance through performance and psychophysiological feedback, is presented. With this setup, the perceived challenge can be modulated by means of the game difficulty and the perceived skill by means of the haptic assistance. A new metric (*FlowIndex*) is proposed to numerically quantify and visualize the challenge/skill relation. The results are contrasted with comparable previously published work and show that the new method afforded a higher *FlowIndex* (i.e., a superior challenge/skill relation) and an improved balance between augmented performance and user satisfaction (higher level of valence, i.e., a more enjoyable and satisfactory experience).

## Introduction

### Human–robot interaction

In recent years, human–robot interaction (HRI) research has mostly focused on a master–slave relationship. However, as a natural step forward, robotics designers have found new and exciting frontiers to expand the existing research toward human-centered fields. Robots are now physically interacting with humans, especially in the medical domain (Colombo et al., 2001; Hochberg et al., 2012; Bonatti et al., 2014). This whole new field of robotics is evolving toward closing the gap between mechatronic systems and humans. In particular, robots have proved to be an excellent research tool in areas of neuroscience such as human motor control (Reinkensmeyer et al., 2004; Huang and Krakauer, 2009) and neurorehabilitation (Daly and Wolpaw, 2008; Hidler and Sainburg, 2011). Besides robots, other emerging technologies, such as virtual reality (Lewis and Rosie, 2012) and haptics (Okamura, 2009), are nowadays in the spotlight of neuroscientists. These new tools allow hacking and augmenting the human visual, auditory, or vibrotactile systems in a controlled way, thus opening up new routes for groundbreaking research.

### Patient-cooperative control

In neurorehabilitation, there are many examples of mechatronic systems designed for human–robot cooperative tasks. The main idea behind these systems is to assist the subject in completing a given task (e.g., reaching or grasping), typically by using a haptic controlled robotic device to deliver the forces needed for the correct completion of the objective (Liu et al., 2006; Nef et al., 2009; Song et al., 2013; Squeri et al., 2014; Pignolo et al., 2016). The intended output of these therapeutic robots is to promote engagement, human effort, and intention of movement and, therefore, to accelerate motor learning and induce neural plasticity (Reinkensmeyer et al., 2004; Edgerton et al., 2008; Huang and Krakauer, 2009; Pennycott et al., 2012). The control strategies of interactive robotic devices are typically designed based on the paradigm “assistance-as-needed,” where human and robot cooperate to successfully complete a task, minimizing the intervention from the robotic device and maximizing that of the human. This patient-cooperative control is mostly based on performance measures such as kinematics, kinetics, muscle activity, etc.

Yet, in spite of human–robot cooperative tasks being “performed” accurately and successfully through these patient-cooperative control paradigms, patient outcome and recovery did not improve significantly with the introduction of “patient-in-charge” robotic rehabilitation devices. Issues such as emotional and cognitive stress, excessive physical work demand, discomfort, pain, boringness, and lack of motivation are rarely taken into account, yet play a key role in human motor control performance (Guadagnoli and Lee, 2004). For example, emotions such as anxiety, frustration, or stress can have a large impact on motor performance, speed, and variability (Coombes et al., 2006). As early as 1908, Yerkes and Dodson (1908) showed that human performance changes in relation to the level of arousal depending on the difficulty of the task: for a difficult task, high levels of arousal can be counterproductive, but if the task lies within the subject's capabilities, high levels of arousal can be beneficial instead (Yerkes and Dodson, 1908; Diamond et al., 2007).

According to Mihály Csíkszentmihályi (1992), cognitive states (e.g., focused, bored, motivated) can also change in relation to the challenge and skill level perceived by a given subject. These cognitive states can either increase or decrease performance accordingly. For instance, a state of boredom will tend to decrease performance, while a focused cognitive state will lead to better results by promoting mental engagement (Guadagnoli and Lee, 2004; Holden, 2005).

### Assessment of emotion

Emotions are accompanied by a set of somatic responses associated with autonomic nervous system (ANS) activity. For a specific emotional state, there exists a probable set of somatic and ANS outputs, although it remains unclear which kind of emotion is associated with which exact autonomic signature (Kreibig, 2010). Nevertheless, psychophysiology has been used to indirectly measure ANS-related responses to external stimuli that affect a person's mood and engagement in a variety of interactive scenarios (Kreibig, 2010). Autonomic responses of emotion are present in parameters such as heart rate (HR), skin temperature (SKT), galvanic skin response (GSR), and respiration rate (RR). A complete overview of these parameters has been summarized in a tag cloud based on 134 publications and explained in detail in Kreibig (2010).

### Biocooperative control

In an attempt to harmonize existing mechatronic systems with human beings, physiological computing has appeared as an enabling technology to give machines exteroceptive capabilities to measure and record physiological data on the emotional state of the user, allowing proactive and implicit adaptations of HRI in real time (Fairclough, 2009, 2011). This concept includes the human in the control loop and gives birth to a whole new set of possible biomechatronic devices capable of “biocooperative adaptation” (Pope et al., 1995; Prinzel et al., 2000).

In 2008–2009, the first ideas of using ANS-related signals as feedback information to close a biocooperative control loop took shape (Bonarini et al., 2008; Mihelj et al., 2009; Novak et al., 2009; Riener et al., 2009; Rodriguez Guerrero et al., 2009). In 2010, Novak et al. (2010a,b) attempted to determine which psychophysiological responses provide the most reliable information about the subject's psychological state during an upper-limb virtual reality (VR) task with the HapticMaster robot in healthy subjects (Novak et al., 2010a) and stroke patients (Novak et al., 2010b; Goljar et al., 2011). Koenig et al. (2010, 2011b) took this research a step further and determined the most suitable psychophysiological parameters to estimate the psychological state of subjects and neurological patients during walking in a driven gait orthosis Lokomat while completing a cognitively demanding VR task (Koenig et al., 2010, 2011e). Also in 2010, Rodriguez Guerrero et al. (2010) presented a step forward toward a working prototype of a biocooperative closed loop that included HR feedback to modulate a set of assistive forces in a robotic-aided neurorehabilitation scenario. Soon after, Novak et al. (2011a,b), Koenig et al. (2011a), Badesa et al. (2012, 2014), and Morales et al. (2014) implemented a more complex closed-loop system that included feedback parameters related to HR, RR, GSR, and SKT (Novak et al., 2011a,b; Koenig et al., 2011b; Badesa et al., 2012, 2014; Morales et al., 2014). These studies aimed to change the difficulty level of the VR task depending on the emotional and cognitive load experienced by the subjects (Koenig et al., 2011b; Novak et al., 2011a,b; Badesa et al., 2012, 2014; Morales et al., 2014). In a previous work, Rodriguez Guerrero et al. (2013) developed a closed biocooperative loop with an auto-tuning fuzzy logic classifier to modulate the haptic assistance given to subjects using HR and GSR feedback. Notice that in contrast with the work by Koenig and Novak (Koenig et al., 2011b; Novak et al., 2011a,b), the goal was not to adapt the game difficulty and thus the perceived challenge level, but to adapt the level of assistance given, thereby attempting to affect the perceived skill level instead. Although the conclusions of Rodriguez Guerrero et al. have suggested that psychophysiological feedback alone can help improve the user experience, independent of whether there exists contextual information about the performance or not, they also suggested that the biocooperative system could be further improved by blending the haptic modulation together with adaptations in game difficulty (Rodriguez Guerrero et al., 2013).

### Article contribution

The Yerkes and Dodson model (Yerkes and Dodson, 1908; Diamond et al., 2007) suggests that arousal levels have a strong impact on performance. It also suggests that high levels of arousal can be counterproductive, especially when the presented task becomes overwhelming to the subject. In contrast, if the task lies within the subject's capabilities, high levels of arousal can be beneficial instead. The contribution of this work expands upon this logic and improves the biocooperative architecture presented by Rodriguez Guerrero et al. (2013) by augmenting the system with contextual performance information that, when blended with the individual's psychophysiological feedback, provides a superior and more individualized continuous adaptation of the system to keep the task difficulty within its user's capabilities, improving subject performance and more importantly the overall user experience. Moreover, as there is still controversy on which psychophysiological parameters are eligible for use in biocooperative enhanced systems, the present study will also take into consideration, as was previously done by Novak et al. (2010a) and Knaepen et al. (2015), which psychophysiological parameters are the most suited for use in a closed biocooperative loop. Finally, a new metric (i.e., *FlowIndex*) is proposed to numerically quantify and visualize the challenge/skill relation.

## Materials and methods

### Subjects

Eleven subjects participated in this study involving an open-loop and a closed-loop experiment. The subjects had no clinical records of neural or motor deficiencies. Six subjects [five males and one female, mean age 30.5 years (*SD* = 3.83)] participated in the open-loop experiment and 11 subjects [eight males and three females, mean age 35 years (*SD* = 7.34)] participated in the closed-loop experiment. All experimental procedures were performed according to the standards set by the Declaration of Helsinki for medical research involving human subjects. Upon arrival in the lab, subjects signed a written informed consent form. This research was approved by the medical ethics committee of Fundación CARTIF.

### Instrumentation

#### Virtual reality game

The virtual scenario was based on a popular “catch the falling droplet” reaching game (see Rodriguez Guerrero et al., 2013 for more details), which has also been used by other robotic rehabilitation platforms such as Armeo (Merians et al., 2009; Schwickert et al., 2011; Wittmann et al., 2016). The game interface and its mechanics are very simple and demand basically no explanation or extra human intervention. Droplets were spawned one at a time at a given velocity, *v*_droplet_, defined in pixels/sec. Only one droplet was rendered at a time, to avoid pre-emptive maneuvers from the subject and maintain focus on the actual target. The subject was expected to catch the falling droplet by moving the PHYSIOBOT end effector, which was represented by a cup in the VR environment. Movements of the robot were projected back to the screen, thereby acting as a visual feedback. The initial positions of the droplets were predefined and generated in advance as an array of 100 positions. This array size is sufficiently large to prevent subjects from memorizing the positions, therefore avoiding habituation issues. The difficulty level of the task could be changed by altering either the distance between droplets or the falling speed. However, by keeping the same initial positions, the total amount of power needed for the task to be successfully completed in a certain amount of time was homogeneous among sessions with a defined velocity (i.e., experiments in the open loop). Therefore, the difficulty modulation was reduced to make changes in the velocity of the falling droplets only. The faster the droplets fall, the greater the amount of power needed to catch them in time, due to the permanent velocity-dependent force field rendered by the haptic device.

#### Haptic assistance

The PHYSIOBOT is capable of rendering a wide range of 3D forces and primitives such as haptic walls, dampers, springs, textures, or a combination thereof (Rodriguez Guerrero et al., 2009). In this setup, a viscoelastic force tunnel was rendered so that it created resistive forces in the sagittal and vertical axes. On the task axis (i.e., transverse axis), a constant-viscosity force field (150 Ns/m) was rendered, such that velocity-dependent resistive forces made the physical task of moving the virtual cup a demanding exercise without the appropriate haptic assistance. The robot could also help the subject by applying assistive forces in the task direction. Forces could be modulated, placed, or removed online at any time.

Optimally, haptic assistance should be computed as the force needed for the robot to take the subject to the target with minimum intervention, and simultaneously promote engagement and self-initiated movements. Therefore, the assistive forces were designed along the task axis, as a critically damped second-order system. A critically damped response allows the system to marginally get to the target in time with the minimum amount of assistance needed to complete the task (see Rodriguez Guerrero et al., 2013 for further details of this calculation). Besides the actuation strategy in terms of magnitude, the timing of the assistive forces needed to be determined. Most of the existing physical human–robot cooperative systems, such as those in rehabilitation robotics, render assistive forces to correct erroneous movements with the help of virtual springs. The problem with this method is that it creates the feeling of being helped rather than the feeling of being more capable/in control of moving the virtual object. The haptic assistance in PHYSIOBOT enhances, rather than corrects, the subject's movements by only giving assistance when the subject has initiated the movement in the target direction. The goal is to “hide” the sensation of being helped and to promote the feeling of being more skillful and capable, a very important feature achieved in this design.

#### Biocooperative control

**Figure 1:**
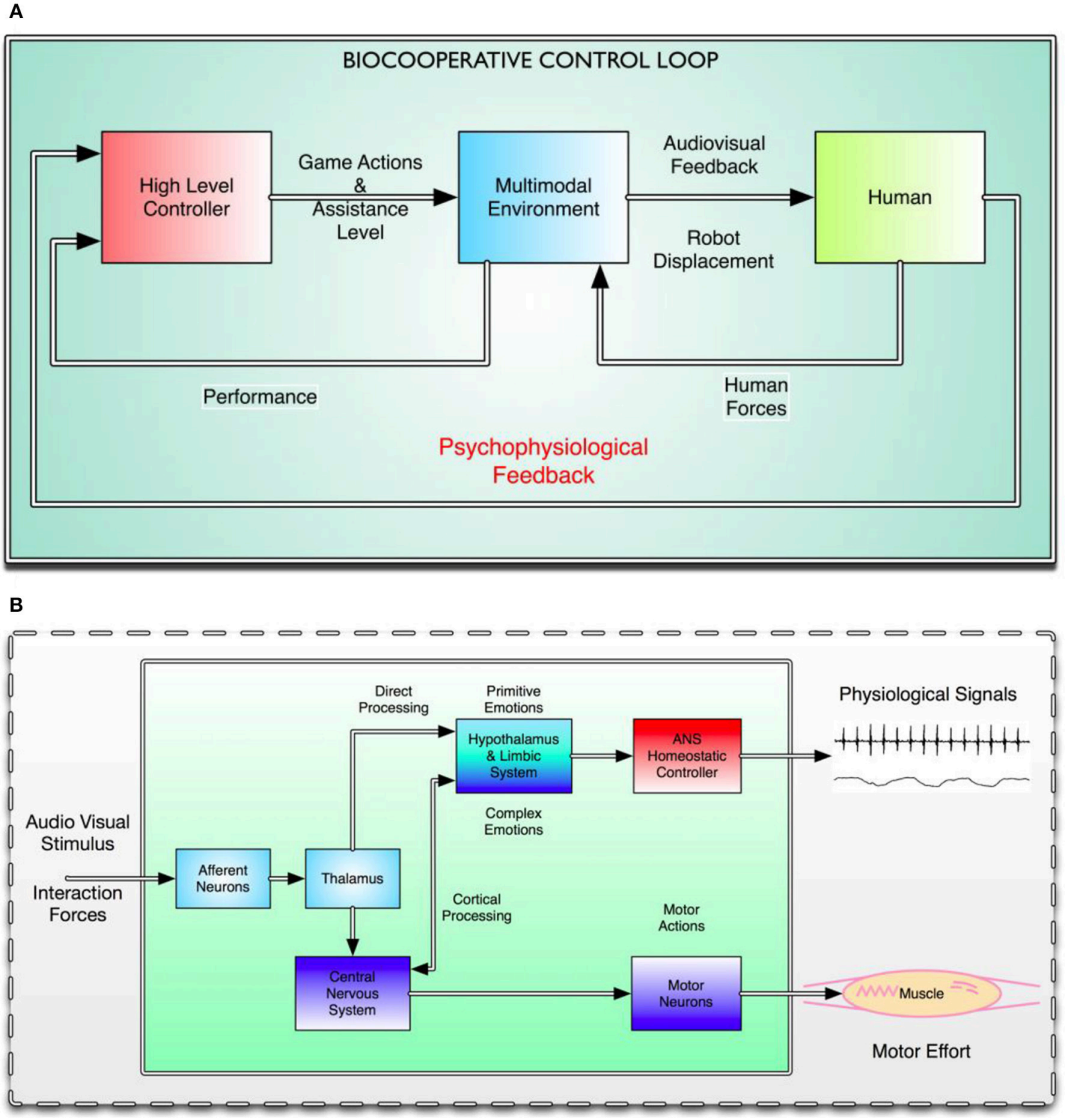
**(A)** Macro view of the biocooperative control loop. Three different interacting blocks can be distinguished. From left to right, the red block represents the high-level controller, which makes decisions based on the overall state of the system, including the subject, and sends actions regarding the difficulty of the game and the level of haptic assistance to the multimodal environment represented by the central blue block. The yellow block represents the human, whose responses to the audiovisual and haptic feedback generate ANS responses and interaction forces, which are, in turn, used to close the biocooperative loop. **(B)** Effects of the multimodal interface (i.e., PHYSIOBOT and VR) on the human control system. External stimuli are captured by the afferent neurons in the peripheral nervous system (PNS) and distributed over different cortical and subcortical levels to the CNS: the thalamus, the limbic system, and the cortex. All external sensory input is received by the thalamus, which sends information simultaneously to the cortex for higher-level processing, and directly to the limbic system (LeDoux, 1995). Part of the information goes to the hypothalamus, which secretes neurohormones that either stimulate or inhibit the secretion of the pituitary hormones that modulate the behavior of the ANS. Simultaneously, the external stimuli make their way to the sensorimotor cortex, which modulates ongoing movements and motor effort, and the prefrontal cortex, which controls the subject's psychophysiological state and turns it into a conscious state and thus a mood or feeling. ANS signals are then fed to the high-level controller and the interaction forces are fed to the multimodal environment as “human in the loop” feedback signals.

In a biocooperative control loop, the human plays a key central role. Figure 1A shows the human as a plant in a classical control scheme. The human block is where the final action lies, and also where the primary feedback is taken from. Figure 1B shows a greatly simplified human control system that only involves some specific portions of the nervous system directly affected by the multimodal interface: the audiovisual stimuli coming from the virtual environment and the interaction forces coming from the physical human–robot interaction with the PHYSIOBOT.

**Figure 2:**
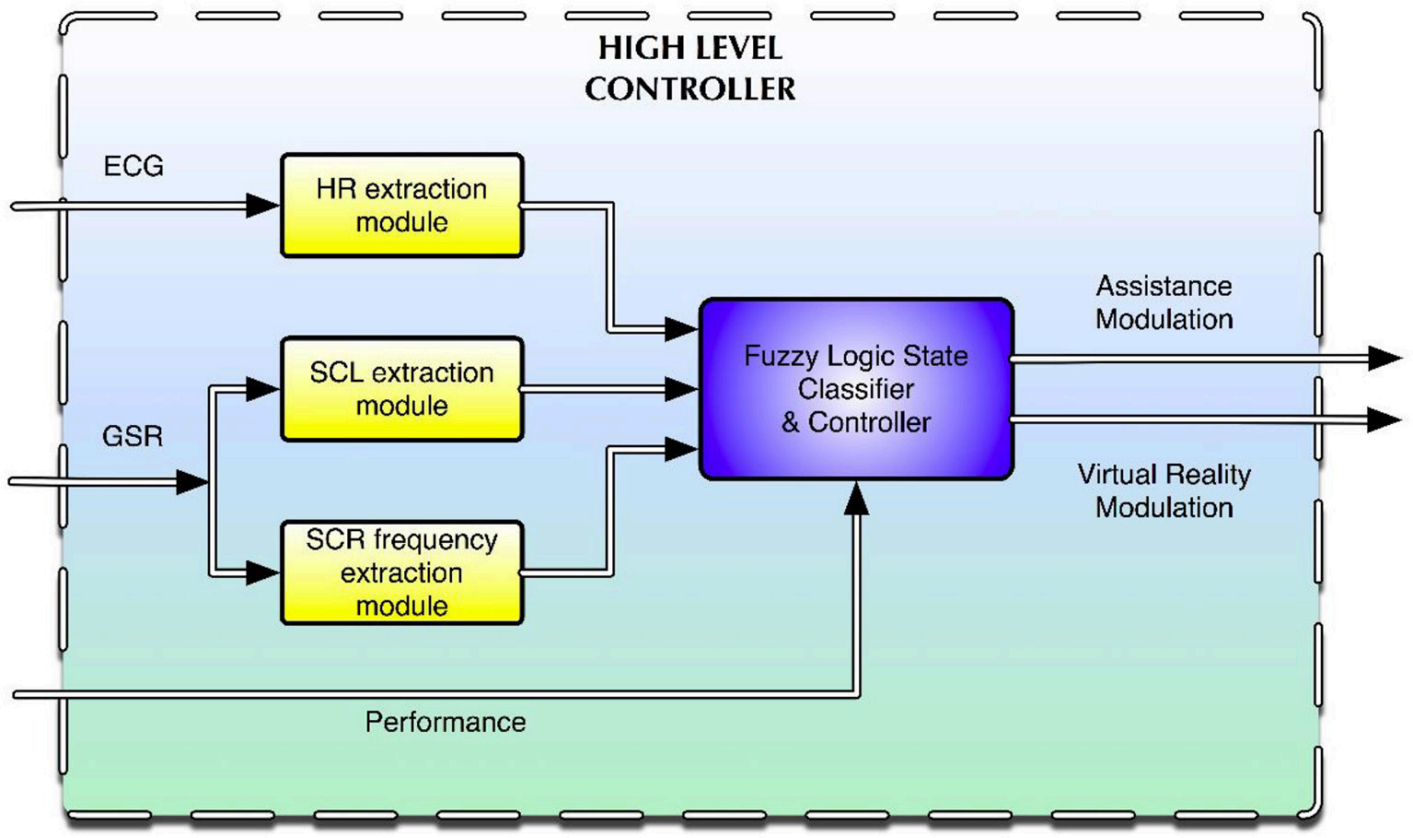
**High-level controller driving the interaction between the subject and the multimodal environment**.

The biocooperative high-level controller used was a Takagi–Sugeno-type fuzzy inference system (FIS) synthesizing several fuzzy rules (see Appendix A in Supplementary material for a complete list of the rules used). Fuzzy logic has already been used in psychophysiological and emotion modeling (Mandryk and Atkins, 2007; Badesa et al., 2012, 2014; Rodriguez Guerrero et al., 2013; Lledo et al., 2015). This system combines data fusion, classification, and control in one simple and well-known framework. The system has four inputs and two outputs (Figure 2).

The results from the open-loop experiments suggested (Section Open-Loop Results: Open-Loop Results) that the most useful signals to use as inputs for the controller were HR and the tonic (i.e., skin conductance level, SCL) and phasic (i.e., skin conductance response frequency, SCR frequency) components of the GSR signal. Performance was the fourth input of the high-level controller. The outputs were the haptic assistance and the changes in game difficulty (i.e., changes in the speed at which the droplets fell). The haptic assistance output was a value between 0 and 1, where 0 represents no assistance and 1 represents full critical assistance. This allows the system to act accordingly when either of the two undesired situations (i.e., under- or overchallenge) occurs for the subject. For instance, if the overall state of the subject indicates that he/she is underchallenged, the assistance will cease, and the difficulty of the task will be augmented accordingly. If, on the other hand, the overall state of the subject indicates that he/she is overchallenged, the assistance will be computed and delivered according to his/her ANS activity, and the game difficulty may be reduced as well.

An intrinsic problem in biocooperative controller design is the inter-/intrasubject variability. In Badesa et al. (2012), fuzzy logic was used but no adaptation algorithm was implemented to deal with this effect. This is important as any consumed substance such as β-blockers, antidepressants, coffee, or sugar may drastically impact baseline ANS readings. In order to deal with the problem of the inter-/intrasubject variability, the FIS was tuned to adapt to the immediate state of each subject. Every FIS has two basic tunable sets of parameters, the rules base, and the membership functions (MFs) for each I/O. The MFs were automatically calibrated after an open-loop “calibration task” designed primarily for that purpose. In the 3-min non-haptic-assisted VR calibration task, the difficulty level was adapted such that every time the subject caught a droplet, the system augmented the falling speed by 2 pixels/s; on the contrary, if the subject missed a droplet, the system reduced the speed by the same amount. The idea behind the calibration task was that the subject's score would steadily increase over time until the challenge level started to approach his/her skill level. After reaching this tipping point, the subject typically started to get drastically aroused as their skill limit was constantly being challenged by the difficulty of the game. The data obtained at the end of the calibration task can be used to tune the MF of the HR, SCL, and SCR frequency. We refer the reader to Rodriguez Guerrero et al. (2013) for more details regarding MF auto-tuning. Besides these two parameters, the rules base and the MFs, a performance parameter (i.e., based on the subject's performance during the VR game) was used in the high-level controller of the biocooperative feedback loop.

**Figure 3:**
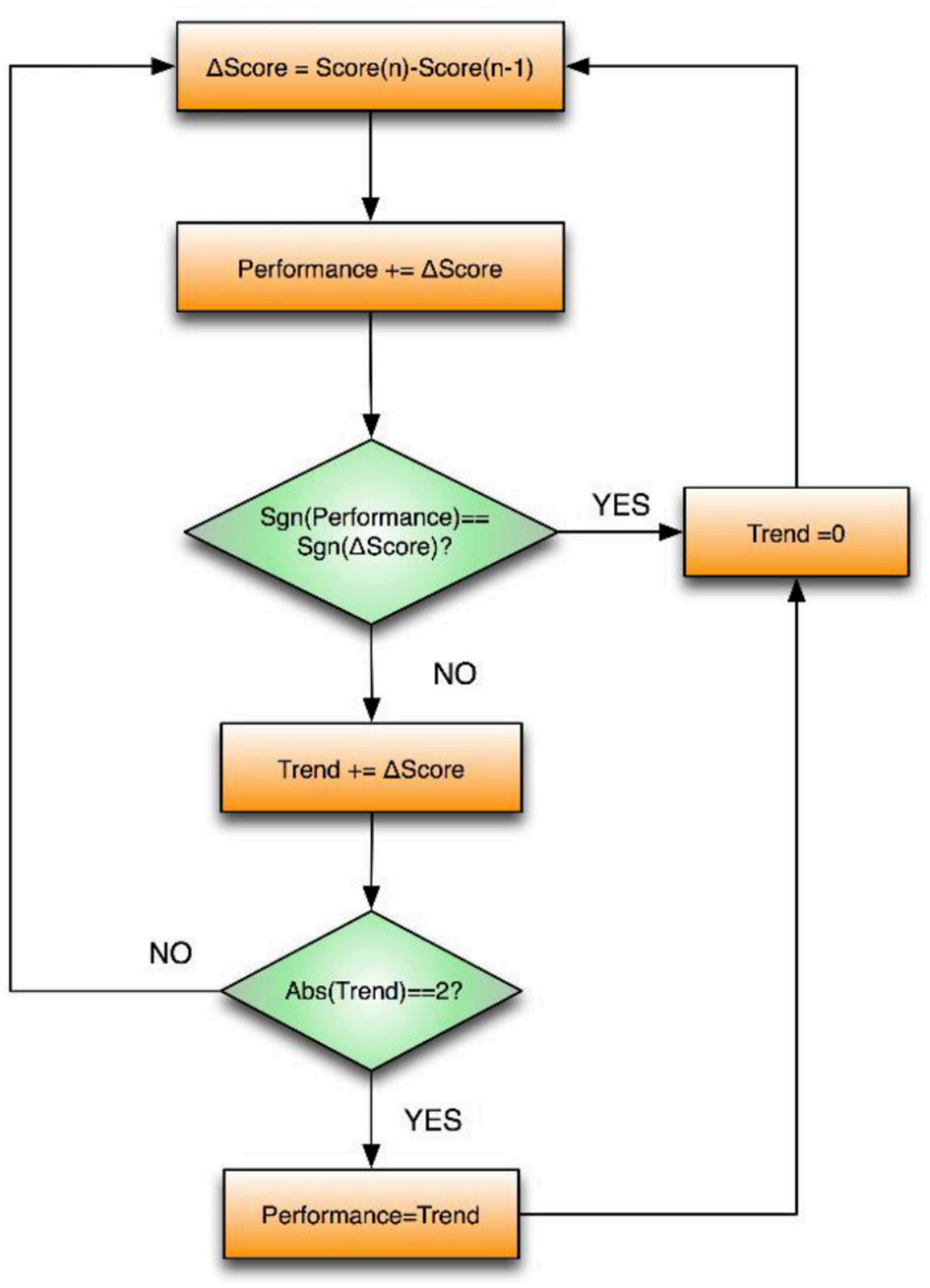
**Schematic overview of the logic for calculating the performance trend in the VR game**.

The algorithm shown in Figure 3 was used to compute the “performance” input to the fuzzy controller based on the trend of the global score. The idea behind this design was that, no matter how well subjects were performing globally (i.e., their total game score), small sustained changes in performance would always influence their emotions. A “trend” or winning/losing streak will often boost or deteriorate our confidence (i.e., dominance value). Therefore, if two droplets were caught/missed in a row, the performance sign changed accordingly in order to take preemptive actions in the controller logic to avoid frustration.

The fuzzy controller worked at a sample rate of 100 Hz, although both of its outputs (i.e., haptic assistance and game difficulty) were updated every epoch (n; the period between two consecutive droplets). Thus, the output of an epoch (n) was the result of the average of the calculations made in the epoch (n−1). As such, the calculations and effects of the inputs that manage the signals were synchronized with the performance input, which was updated every epoch (n). This averaging contributes to filtering the outputs, especially the haptic assistance, so that the subject does not feel as though it is changing continuously, thereby producing a more natural, less noisy feeling of the haptic assistance and avoiding a constantly varying droplet speed.

### Experimental design

Upon arrival in the lab, the purpose and procedure of the experiment were explained and the subjects signed the written informed consent form and were equipped with the Biopac MP150 data acquisition system for measuring ANS responses (i.e., ECG, GSR, and SKT). Next, subjects participated in two different experiments involving a VR task of catching falling droplets by manipulating the PHYSIOBOT end effector (see Rodriguez Guerrero et al., 2013 for a picture of the experimental setup). During each session, electrophysiological data, game performance, and physical performance were measured, and immediately following each session, the emotional status of the subject was registered.

#### Open-loop experiment

The first experiment was an open-loop experiment (*n* = 6) consisting of a warm-up of 3 min followed by four game sessions of 5 min each. The 3-min warmup time (i.e., no physical or mental exercises were performed) was used to stabilize the sensor readings to a baseline. The four experimental sessions contained four different levels of difficulty ranging from very easy to very hard, starting with difficulty level 3, then difficulty 4, followed by difficulty 2, and finally difficulty level 6 (Table 1). All difficulty levels were chosen in advance, prior to the experiment.

**Table 1:** **The four levels of task difficulty in the open-loop experiment (*n* = 6)**.

**Difficulty level**	**Description**	**Presentation order**
2	*Very easy*: This task is meant as a baseline period. It maintains the subject's focus and helps to reduce psychophysiological responses elicited by random mental thoughts or memories.	3rd
3	*Normal:* This task makes a good transition between the baseline period and the following trials.	1st
4	*Challenging:* This is a more demanding task than level 3, but in general, it fits better with the average subject preferences.	2nd
6	*Very hard:* Although this level can be quite hard, it is not designed to be frustrating but demanding instead. It is meant to force the subject into an aroused state.	4th

#### Closed-loop experiment

The second experiment was a closed-loop experiment (*n* = 11) consisting of a control task of 5 min (i.e., the calibration task, hereafter referred to as CT), followed by a 5-min resting period and a 5-min closed-loop trial (hereafter referred to as haptic and difficulty modulation (HDM) controller). The obtained data were compared to the results obtained in Rodriguez Guerrero et al. (2013), where a haptic-assisted-only controller was used (hereafter referred to as HAO).

### Data acquisition and analysis

#### VR score

The score on the VR task was calculated based on the game score, which added one point for each droplet caught and subtracted one point if a droplet was missed. The score mean value with standard deviation (SD) is presented for each open- and closed-loop experiment in the results.

#### Physical performance

Physical performance was computed by measuring the interaction force with the PHYSIOBOT haptic device by means of a force sensor mounted between the end effector and the human. The mean absolute force (MAF) expressed in newtons (N) with SD was used as an indicator of physical performance and was computed by extracting the mean of the absolute value of all of the forces over the task axis during a session, sampled at a rate of 1 kHz.

#### Electrophysiological assessment

For the physiological signal recording, the Biopac MP150 data acquisition system^1^ with three amplifiers, ECG100c^2^, GSR100c^3^, and SKT100c^4^, was used. All recordings were collected online during the game time through UDP/IP at a frequency of 100 Hz. All physiological measurements were recorded through non-invasive, ambulatory sensors, using simple, and fast Velcro attachment electrodes for GSR and SKT and sticky disposable electrodes for the ECG. The mean values for the 5-min experimental sessions (i.e., open- and closed-loop experiments) were calculated. From the ECG, the intervals between two heartbeats (NN intervals) were extracted in order to calculate the mean HR value with SD. Two components were extracted from the GSR signal: SCL and SCR frequency. The mean SCL with SD, which is the baseline level of skin conductance, was calculated for each game difficulty level. The SCR frequency represents increases in skin conductance followed by a return to the tonic level. The mean SCR frequency with SD as well as mean SKT with SD are presented in the results.

#### Emotion assessment

To subjectively assess emotion, the self-assessment manikin (SAM) test was used (Posner et al., 2005, 2009) (see Appendix A in Supplementary Material). We implemented a computer program that presented the SAM test at the end of each session in an automated way, such that the subjects were able to select their choice without any external human intervention that might have otherwise biased their answers. The three emotional dimensions used were arousal, valence (i.e., pleasure), and dominance (i.e., sense of control of the situation). Each dimension was rated on a nine-point scale (i.e., 1–9). For each dimension, the mean value with SD is presented.

#### Vector representation, *FlowIndex*, and *FlowDir*

In this article, a new and simple set of tools is proposed for analyzing, visualizing, and comparing the impact of a biocooperative controller based on arousal and dominance scores: the vector representation, *FlowIndex*, and *FlowDir*. These vectors are tools to graphically and numerically evaluate the results of a given control strategy designed to act on the challenge/skill perception of a particular task.

**Figure 4:**
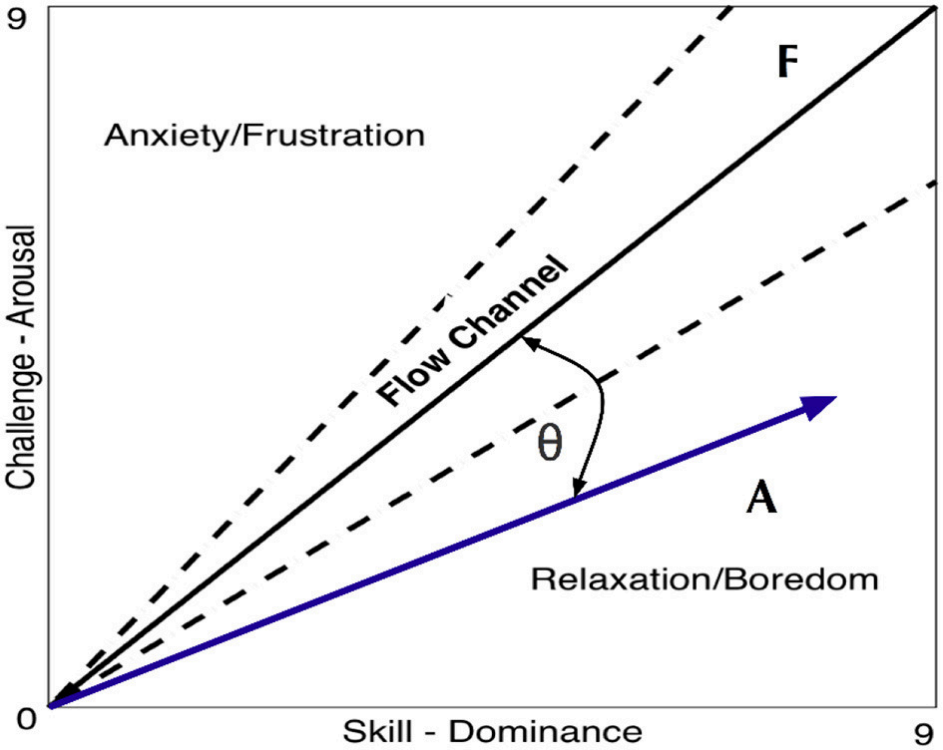
**Vector F is the reference with components [dominance, arousal] = [9,9] and vector A is used as an example of a given output obtained within a session with coordinates [8,5]**. The vector representation affords a quick glance into the performance of a given session. In this example, the results point toward the relaxation/boredom zone of the figure and therefore it is unlikely that the experience was enjoyable or engaging but rather boring instead.

The vector representation (Figure 4) is a visual way to present the challenge/skill ratio and compare the [dominance, arousal] results (i.e., from the SAM questionnaire or as a result of the output of an automatic classifier) of one or more tasks (i.e., vector **A**) relative to the maximal/optimal challenge/skill ratio (i.e., reference vector **F**). This metric is a mathematical representation inspired by a theory used in psychology termed “*Flow* statem,” where an optimal experience motivates people to further learn or stay committed to a task (Csikszentmihalyi and LeFevre, 1989; Engeser and Rheinberg, 2008). The challenge level relates to the arousal domain and can be modulated by means of the game difficulty level, and the skill level is related to the dominance domain and can be modulated by means of the haptic assistance; see Equation (Hochberg et al., 2012). Therefore, the equivalence for their ratios can be defined as:

(1) $$\frac{Challenge}{Skill}\;≅\;\frac{Arousal}{Dominance}$$

Since this relation has two components, it can be expressed and plotted as a vector (Figure 4).

*FlowIndex* uses the obtained pair **A**[dominance, arousal] represented in vector form, and quantifies how alike vector **A** (blue) and the ideal vector **F** (solid black) are (Figure 4). This form of vector similarity first uses the normalized scalar projection (NSP) (Equation 2) of **A** projected onto **F**. The result of this calculation is a scalar bounded between 0 and 1 that gives information on how **A** projects onto **F**. Then, the angular similarity (AS) (Equation 3) is used to calculate the similarity between the two vector angles and gives again a value bounded between 0 and 1 (i.e., where 0 is completely orthogonal and 1 is perfectly parallel). By multiplying the two obtained results (Equation 4), the AS penalizes vectors that are diverging from **F** (i.e., the angle between them is >0). Notice that both NSP and AS are bounded between 0 and 1 and, therefore, the *FlowIndex* results will always be bounded in the same way. The *FlowIndex* is then a scalar value that represents how alike **A** and **F** are in terms of both angle and magnitude.

The higher the *FlowIndex* value is, the closer we are to being within the flow channel (i.e., the dotted lines delimiting the ideal range of challenge/skill ratio in Figure 4) and, therefore, the more pleasant and exciting the experience will be.

(2) $$NSP=\frac{A.\hat{F}}{\left||F|\right|}$$

(3) $$AS\;=\;cos\theta \;=\left(\frac{A.F}{\left||A||||F|\right|}\right)$$

(4) FlowIndex=NSP∗AS

Since *FlowIndex* is always positive and bounded between 0 and 1, this metric alone cannot reveal if the resulting vector is pointing toward the frustration or relaxation zone. Nevertheless, the direction of the *FlowIndex* can be calculated. This computation is straightforward and can be performed as follows:

(5) $$FlowDir=\{\begin{array}{ccc} Frustration\;sign\left(A_{y}-A_{x}\right) & = & 1\\ Flow\;sign\left(A_{y}-A_{x}\right) & = & 0\\ Relaxation\;sign\left(A_{y}-A_{x}\right) & = & -1 \end{array}$$

Equation (5) gives a value of 0 whenever vectors **A** and **F** are parallel (i.e., *flow*), 1 whenever vector **A** is pointing toward the frustration zone, and −1 whenever it is pointing toward the relaxation zone. For this given example, the *FlowIndex* of **A** = [8,5] is 0.65 with *FlowDir* = *Relaxation*.

### Statistical analysis

Statistical analysis was performed using the Statistical Package for Social Sciences 24.0 for Windows statistical software (SPSS Inc., Chicago, IL, USA). Data are presented as mean with SD. Statistical significance was accepted at *p* < 0.05. As *n* = 6, non-parametric statistics were applied and effect sizes were calculated. Eight variables were extracted from all recorded data: valence, dominance, and arousal were extracted from the SAM; MAF from the haptic device; and SKT, HR, SCL, and SCR frequency were derived from physiological recordings. To assess differences between the four difficulty levels in the open-loop experiment (*n* = 6), a Friedman's analysis of variance (i.e., ANOVA) was used. As an estimate of effect size, Kendall's *W* coefficient was calculated, where a value of *W* = 0 indicates no relationship and a value of *W* = 1 indicates a perfect relationship (Tomczak and Tomczak, 2014). The following null hypothesis was tested, for which μ_*i*_ is the mean of the observed variable for each difficulty level (i.e., subindex *i* corresponds to difficulty levels 2, 3, 4, and 6): no significant differences between the four difficulty levels, H_0_: μ_2_ = μ_3_ = μ_4_ = μ_6_.

Furthermore, Spearman's rank correlation coefficient was used to measure the strength of the association between the subjective data from the SAM questionnaire and objective data such as HR, SCL, SCR, SKT, and MAF.

## Results

### Open-loop experiment

**Figure 5:**
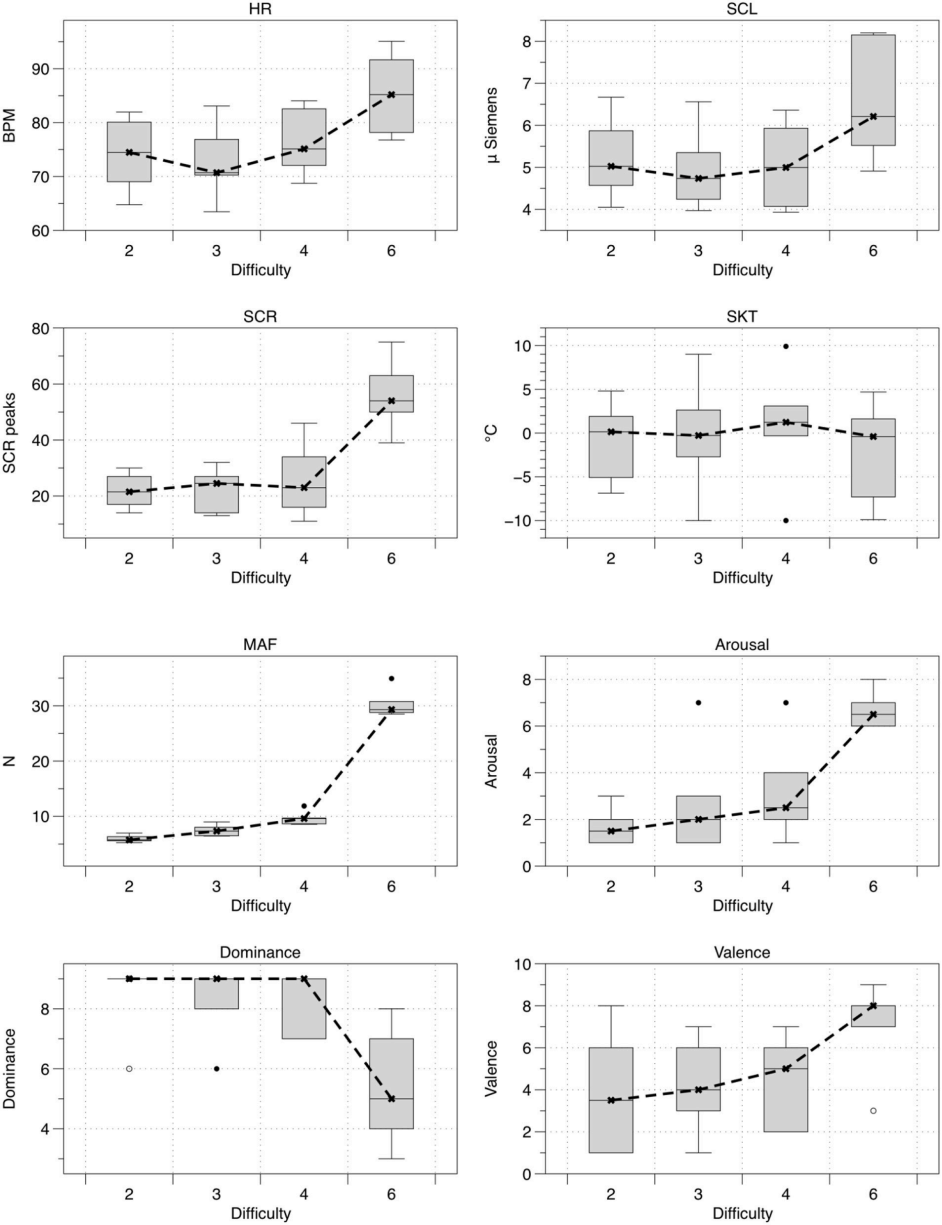
**Boxplots with mean values of HR, SCL, SCR frequency, SKT, MAF, arousal, dominance, and valence for the four levels of game difficulty (*n* = 6)**. The shaded boxes represent the range in which 50% of the data fell (i.e., interquartile range). The “I” shaped whiskers represent all of the data that fell within 3 SD of the mean (i.e., the black horizontal bars). The open (◦) and closed (•) circles above the whiskers represent outliers (i.e., individual scores very different from the overall scores in the shaded box). Difficulty is presented in ascending order, although the presentation order in the experiments was 3, 4, 2, 6 (see Table 1).

Figure 5 shows the mean values for all of the eight measured variables (*n* = 6) in the order of difficulty i.e., 2, 3, 4, and 6 (note that the difficulty levels were presented to the subjects in a different order; see Table 1). Table 2 shows the outcome of all eight variables for the tested hypothesis (H_0_) with the corresponding effect size. Significant differences (*p* < 0.05) and large effect sizes between all four difficulty levels were found for arousal, dominance, MAF, SCR frequency, HR, and SCL (Figure 5 and Table 2).

**Table 2:** **Significant differences and effect sizes between game difficulty levels for the eight measured variables**.

	**H_0_**	**Effect size *W***
Arousal	*p* = 0.001^*^	*W* = 0.883
Dominance	*p* = 0.002^*^	*W* = 0.818
Valence	*p* = 0.125	*W* = 0.319
MAF	*p* < 0.001^*^	*W* = 1.00
SCR frequency	*p* = 0.006^*^	*W* = 0.698
HR	*p* = 0.002^*^	*W* = 0.833
SKT	*p* = 0.642	*W* = 0.093
SCL	*p* = 0.008^*^	*W* = 0.656

* *Significant difference, p < 0.05*.

The results of the Spearman's rank correlation analysis between the subjective variables of arousal, dominance, and valence, on the one hand, and objective psychophysiological responses (i.e., HR, SCL, SCR, SKT) and physical effort (i.e., MAF), on the other hand, can be found in Table 3.

**Table 3:** **Correlations between subjective and objective measurements**.

	**HR**	**SCL**	**SCR**	**SKT**	**MAF**
Arousal	*p* = 0.221	*p* = 0.003^*^	*p* = 0.011^*^	*p* = 0.110	*p* < 0.001^*^
	*r* = 0.259	*r* = 0.572	*r* = 0.512	*r* = −0.334	*r* = 0.708
Dominance	*p* = 0.807	*p* = 0.001^*^	*p* < 0.001^*^	*p* = 0.414	*p* = 0.004^*^
	*r* = −0.053	*r* = −0.620	*r* = −0.681	*r* = 0.175	*r* = −0.560
Valence	*p* = 0.525	*p* = 0.044^*^	*p* = 0.061	*p* = 0.706	*p* = 0.044^*^
	*r* = −0.136	*r* = 0.415	*r* = 0.388	*r* = −0.081	*r* = 0.415

* *Significant correlation, p < 0.05*.

### Closed-loop experiment

**Figure 6:**
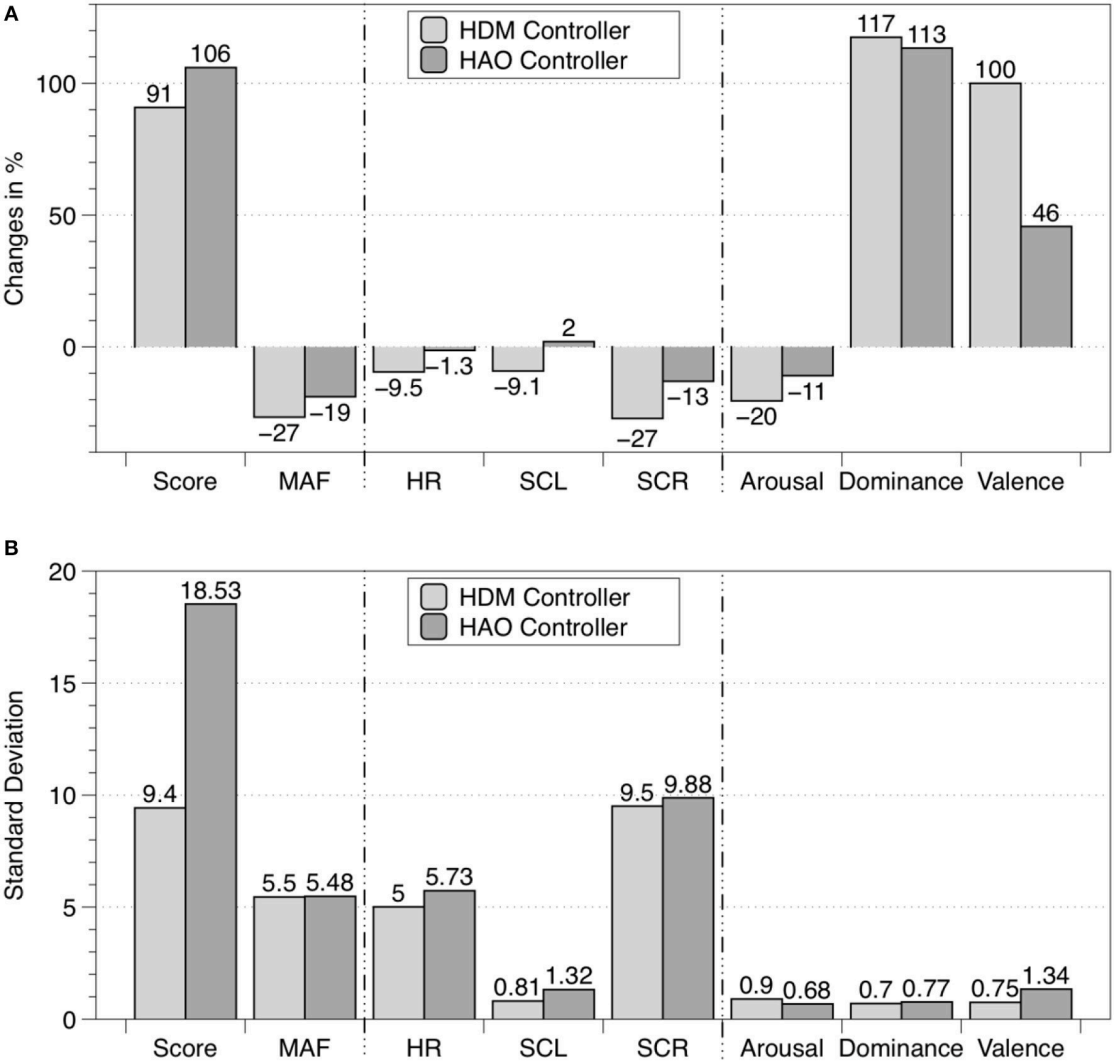
**(A)** Percentage change between baseline (i.e., calibration task) and the closed-loop experiments for the eight measured variables. **(B)** The corresponding standard deviations. Light gray bars represent the data obtained for the HDM controller described in this article, and dark gray bars represent data from the HAO controller published in Rodriguez Guerrero et al. (2013). In both cases the sample sizes were *n* = 11.

Table 4 shows the mean values with SD for the total game score, MAF, HR, SCL, SCR frequency, arousal, dominance, and valence during the 5-min calibration task and the 5-min closed-loop task. The percentage change between the results of the closed-loop experiment and the baseline, i.e., the calibration task, are presented in Figure 6.

**Table 4:** **Mean values with SD for the eight measured variables during the 5-min calibration and 5-min closed-loop tasks for the HDM and HAO controller**.

**Variable**	**Unit**	**CT_HDM**	**HDM**	**CT_HAO**	**HAO**
Total game score	Points	29.72 ± 2.68	56.72 ± 9.43	30.09 ± 2.11	62.63 ± 18.53
MAF	N	34.45 ± 5.92	25.27 ± 5.45	34.27 ± 5.85	27.82 ± 5.48
HR	BPM	88.99 ± 5.24	80.55 ± 5.01	87.75 ± 5.91	86.57 ± 5.73
SCL	μS	6.56 ± 1.11	5.96 ± 0.81	6.4 ± 1.17	6.52 ± 1.32
SCR frequency	Counts per task	67.27 ± 8.24	49 ± 9.51	67.27 ± 9.88	58 ± 10.16
Arousal	N/A	8.45 ± 0.52	6.72 ± 0.9	8.36 ± 0.67	7.45 ± 0.68
Dominance	N/A	3.72 ± 1.1	8.09 ± 0.7	3.90 ± 1.70	7 ± 0.77
Valence	N/A	4.09 ± 1.37	8.18 ± 0.75	4.54 ± 1.63	6.27 ± 1.34

*CT_HDM, calibration task in HDM experiment; HDM, experiment with haptic and difficulty modulation controller described in this paper; CT_HAO, calibration task in HAO experiment; HAO, haptic-assistance-only experiment from 2013 (Rodriguez Guerrero et al., 2013); MAF, mean absolute force; HR, heart rate; SCL, skin conductance level*.

## Discussion

### Open-loop results

For a psychophysiological signal to be useful as feedback information for control purposes, it has to change considerably and coherently in relation to the task difficulty. From the results shown in Table 2, we can infer that arousal, dominance, MAF, SCR frequency, HR, and SCL changed significantly across the four difficulty levels (i.e., *p* < 0.05). These results are coherent with previous studies on psychophysiological changes during mixed physical/psychological HRI tasks (Rodriguez Guerrero et al., 2010; Novak et al., 2010a,b, 2011a,b; Koenig et al., 2011a,c; Badesa et al., 2012, 2014; Morales et al., 2014) and show that, in this experiment, HR, SCL, and SCR were the most sensitive physiological parameters to discriminate between the difficulty levels of the VR task. For HR, the observations made went further and showed that this variable exhibited a considerable inertia when preceded by a higher level of physical activity, as can be seen in Figure 5 (i.e., delayed effect of task presentation order, see Table 1; more specifically, a continued effect of level 4 on level 2 can be seen). This delay in HR decrease during recovery from a higher level of physical effort is a well-known effect (Shetler et al., 2001; Vijayalakshmi et al., 2014). In healthy subjects, a decrease of 15–20 beats per minute in the first minute of recovery has been shown to be typical (Shetler et al., 2001). This slower vagal reactivation may have to be taken into account in future research, as such inertial effects may become important when the subject is exposed to a wider range of physical loads over an extended period of time. In such cases, adding an appropriate corrective factor to the HR might be beneficial. The SCL and SCR behaved similarly to HR and increased significantly with increasing level of game difficulty. This is comparable to what has been found in other studies (Novak et al., 2010a; Badesa et al., 2012, 2014; Morales et al., 2014; Knaepen et al., 2015). The SKT was the only physiological variable that did not differ significantly between the four difficulty levels. Although decreases in SKT have been put forward as a marker to detect changes in mental workload (Ohsuga et al., 2001; Koenig et al., 2011e), other studies have found no significant differences for SKT with changes in mental workload (Novak et al., 2010a; Badesa et al., 2012; Knaepen et al., 2015) or found that variations depended on the test subject (Morales et al., 2014). Novak et al. (2010a) proposed that a certain threshold of mental workload should probably be exceeded before the SKT starts to decrease significantly (Novak et al., 2010a). The hardest level (i.e., level 6, see Table 1) in this study was designed to be demanding but not frustrating, and as such this threshold might not have been exceeded, which could explain why the SKT did not change significantly. Moreover, the slow response of the SKT, as also shown by Novak et al. (2010a), makes it difficult to use it in a closed-loop biocooperative scenario. Therefore, we have discarded SKT as a possible feedback signal for the proposed closed-loop controller. As a result, HR, SCR, and SCL proved to be the most useful indicators of psychophysiological activity in a mixed physical/psychological load scenario.

To date, it remains unclear which kinds of emotion are associated with which kinds of objective autonomic signature (Kreibig, 2010). Therefore, objectively measuring emotion through physiological signals remains challenging. In our study, a subjective SAM test was used in order to afford a numerical measurement of perceived emotion. However, it is difficult to integrate a subjective measure of emotion into the control loop of assistive robotic devices. Therefore, we also performed correlations between SAM scores and the other psychophysiological and physical parameters. Results from the Spearman correlation analysis (Table 3) showed that, while HR did not correlate with any of the subjective emotional scores, arousal was positively correlated (*r* > 0, *p* < 0.05) with SCL and SCR. Dominance, on the other hand, was negatively correlated with SCL and SCR (*r* < 0, *p* < 0.05), while valence was only positively correlated with SCL (*r* > 0, *p* < 0.05). These few significant correlations show that it remains difficult to achieve mapping between physiological signals and the emotional state of a subject based on a few physiological measures. The significant correlations between MAF, on the one hand, and arousal, valence, and dominance, on the other hand, further confirm that additional contextual information and probably more physiological parameters are necessary to accurately predict the emotional states of subjects during HRI. This corresponds to what Kreibig (2010) pointed out: as emotions consist of an integrated variety of physiological responses, it is important to select a sufficient number of response measures to allow for the response pattern and its variations to be accurately identified (Kreibig, 2010). Further research will have to carefully consider which additional physiological parameters should be included in the biocooperative controller in order to correctly identify and influence emotional states of the subjects.

### Biocooperative closed-loop control

Results from the SAM questionnaires and *FlowIndex* suggest that the user satisfaction and challenge/skill relation were effectively improved in this work, where haptic and difficulty modulation (HDM) was implemented, compared to the calibration task (CT) and the haptic-assisted-only (HAO) results presented in Rodriguez Guerrero et al. (2013). We invite the reader to take a look at Supplementary Figure 1 (i.e., the SAM scale) in Appendix A, to provide visual support during the upcoming discussion.

#### User satisfaction

The dominance value obtained for HDM (8.09) was superior compared to those obtained for CT (3.72) and for the HAO controller (Hidler and Sainburg, 2011; Table 4). An improved dominance values translates into an improved “perceived skill.” Besides dominance, the valence scores showed the most interesting and desired improvements. The HDM controller scored 8.18 on the valence scale, while values of 4.09 and 6.27 were obtained for the CT and the HAO, respectively. The differences in standard deviation between HAO and HDM for both dominance and valence can be neglected (i.e., 0.07 and 0.59, respectively). The combination of high dominance (i.e., feeling under control of the situation) and high valence (i.e., pleasure) while still being challenged is likely to influence motivation and, thus, to promote future practice and total duration of training, which, in turn, might influence the success of motor learning (Shetler et al., 2001; Guadagnoli and Lee, 2004; Vijayalakshmi et al., 2014).

#### User performance

The results for the total game score obtained with HDM (56.72) were greatly improved compared with the scores obtained for the CT (29.72). However, when compared to the total game score for HAO (62.63), the results ended up being lower by a mean of six points per session. Score variability was expected, as not only the skill but also the assistance given to each subject was different. The assistance given depended entirely on the subject's individual performance and ANS activity, which, of course, is highly dependent on how each subject reacts to pleasant/unpleasant situations. As the spawn frequency of the droplets was also being controlled by the game difficulty, the maximum possible number of points varied with each subject's skill. The more skillful a user was, the more challenging the session would likely be and the more droplets would be rendered. Nevertheless, as the HAO difficulty was not controlled, HDM might be better suited for longer training sessions, as it can provide larger/faster adaptation steps and preemptive maneuvers when fatigue starts to appear, by using the extra information from the performance input.

#### Vector representation, *FlowIndex*, and *FlowDir*

**Figure 7:**
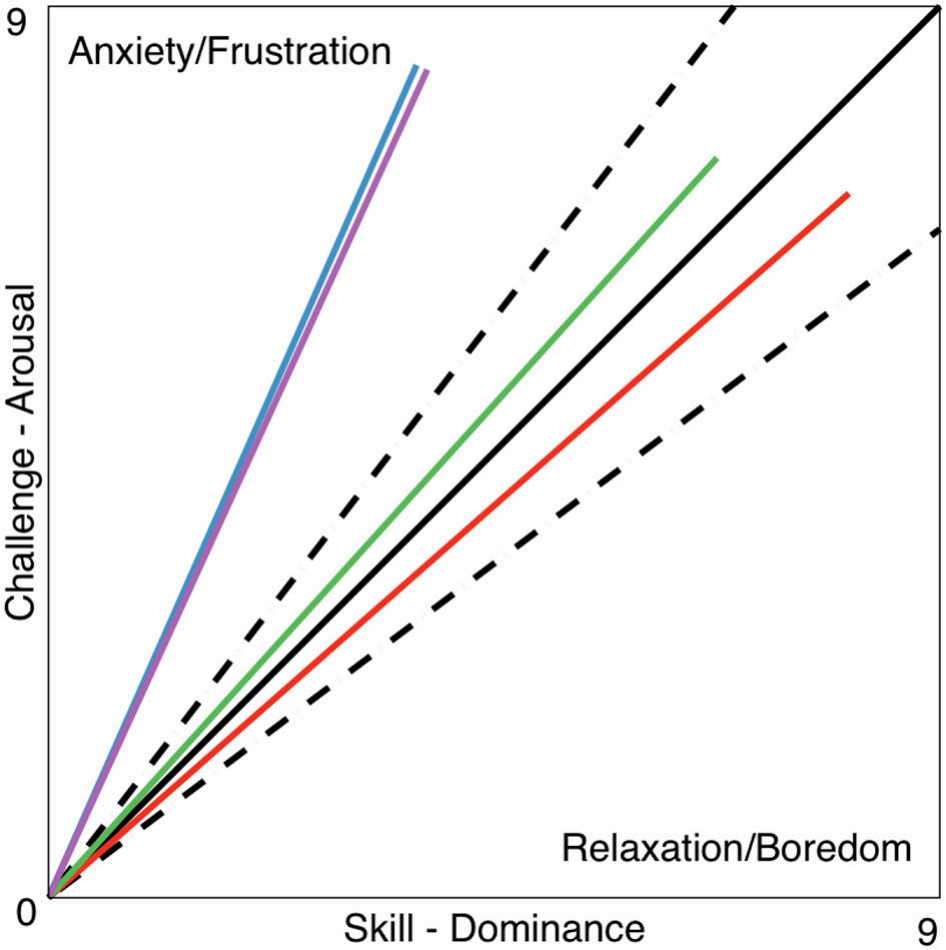
**Vector representation of the challenge/skill relation for the CT _HDM (solid blue line) and HDM (solid red line) tasks**. The data for CT_HAO (solid purple) and HAO (solid green) tasks are also shown. The dotted lines delimit the flow channel, i.e., the range reflecting an ideal challenge/skill ratio, and the solid black line represents the maximal challenge/skill ratio **F** = [9,9]. The presented data is taken from Table 5.

Within the framework of this article, a new set of tools was proposed based on arousal and dominance scores, to indirectly evaluate the impact of a biocooperative controller: the vector representation, *FlowIndex*, and *FlowDir*. These tools allow the graphical and numerical evaluation of the results of a given control strategy designed to improve the perception of a given task. An optimal *flow* level, or optimal challenge/skill ratio, results in the high commitment of a person to a given task (Engeser and Rheinberg, 2008) and also stimulates motor learning (Guadagnoli and Lee, 2004). Previous studies have shown that, for optimal motor learning, the motor task should challenge, arouse, and excite the subject, while not being too stressful or boring (Maclean and Pound, 2000; Guadagnoli and Lee, 2004; Koenig et al., 2011d; Rodriguez Guerrero et al., 2013). The arousal results for HDM (6.72) decreased compared to those for the CT (8.45) and HAO (7.45). Since HDM is able to control the game difficulty (i.e., challenge), arousal can also be greatly influenced by its actions. The decrease in arousal obtained by the HDM controller seems to have played a favorable role for this controller, keeping the *FlowDir* in the *relaxation* zone (Figure 7). This is in contrast with the *FlowDir* for HAO, which was located in the *frustration* zone (Figure 7). HDM also performed better on the *FlowIndex* (i.e., 0.82 vs. 0.80 obtained for the HAO controller; see Table 5), meaning that it was closer to the optimal challenge/skill ratio. Although this difference in *FlowIndex* between HAO and HDM is small, HDM also afforded considerably higher values for valence (Figure 6). It seems that when subjects are close to the *flow* zone, they perform better under the *relaxation* zone rather than under the *frustration* zone (Figure 7). While the evidence for the previous statements is yet to be fully proved, the assumptions are supported by the Yerkes and Dodson model (Yerkes and Dodson, 1908; Diamond et al., 2007), as high levels of arousal (such as those triggered by frustration) can be counterproductive, and the fact that this controller setup led to a more enjoyable experience. Not only did the biocooperative controller presented in this work perform better, it also added greater flexibility to tune the system with an extra degree of freedom by controlling the difficulty of the game. This allows modulation of the challenge/skill relation, with challenge being controlled by the game difficulty and skill by the haptic assistance. This could certainly be exploited in order to fine-tune the system rules and, thus, to maximize the *FlowIndex* and obtain a *FlowDir* closer to the optimal *flow* zone.

**Table 5:** ***FlowIndex*** **and *FlowDir* for the 5-min calibration and 5-min closed-loop tasks for the HDM and HAO controllers**.

	**CT_HDM**	**HDM**	**CT_HAO**	**HAO**
*FlowIndex*	0.63	0.82	0.64	0.8
*FlowDir*	*Frustration*	*Relaxation*	*Frustration*	*Frustration*

*CT_HDM, calibration task in HDM experiment; HDM, experiment with haptic and difficulty modulation controller described in this paper; CT_HAO, calibration task in HAO experiment; HAO, haptic-assistance-only experiment from 2013 (Novak et al., 2011b)*.

### Study limitations

The sample size used here, especially for the open-loop setup (*n* = 6), was small. Therefore, caution should be taken in attempting to generalize the statistical results. The significant differences and large effect sizes indicate an interesting effect that should be confirmed in future studies with a larger sample size and thus a higher statistical power. Although, the difficulty settings for the open-loop trials were satisfactory for the subjects who tested them prior to the experiments, it seems that we could have added another higher level of game difficulty. In this study, we visualized more the effects of the low difficulty range (i.e., game difficulty levels 2, 3, and 4). Only one level of higher game difficulty was present (level 6). The addition of another session with an even higher level of difficulty would have allowed us to more thoroughly explore the effects of the biocooperative controller, as with the difficulty levels used the subjects never really became frustrated and their dominance levels therefore started to decay only at level 6. It would have been useful to explore these effects over longer periods of time to better understand the signal dynamics and compensate for phenomena such as signal inertia. It would also have been useful to investigate and compare the HAO controller to the HDM controller over longer sessions. It is probable that the improved HDM adaptation could have clearer benefits (i.e., higher scores for *FlowIndex* and valence) over longer sessions, where fatigue, boredom, or frustration would be more frequent.

## Conclusion

User satisfaction and augmented human performance are the most important features that any human–machine interface can achieve. The architecture and control strategies presented in this work effectively delivered a well-balanced tradeoff between these key design points. For a human-cooperative controlled interface such as the one presented in this work, augmenting human performance could be as easy as delivering the needed assistance to complete the task. However, this lacks the engagement component that drives motivation and, therefore, it always coincides with the tradeoff of deteriorating user satisfaction. Consequently, a biocooperative interface should ideally deliver individually tailored adaptation based on the user's psychophysiological state and (if available) contextual performance information. Augmenting the system with contextual performance information together with the individual's psychophysiological feedback provides a superior and more individualized adaptation of the system to maintain the task difficulty within its user's capabilities, improving subject performance and more importantly the overall user experience. By modulating both haptic assistance and game difficulty, designers have greater potential to control dominance and arousal levels. This has a direct impact on the capabilities to control the challenge/skill relation of a given task.

Psychophysiological activity under physically demanding scenarios can be measured by using ANS-related signals such as those used in this work. However, the direct mapping against subjective emotional scales like arousal, valence, and dominance seems to remain a challenge that could soon be possible to achieve with modern deep-learning algorithms. Nevertheless, by using psychophysiological feedback in conjunction with additional contextual information (i.e., performance), an overall estimation of the emotional state of a subject can still be made and exploited for control purposes with good results.

Although the given inputs for *FlowIndex* and *FlowDir* are based on the SAM scale, future work needs to be carried out to achieve mapping between arousal and dominance, on the one hand, and psychophysiological signals, on the other hand, so that the calculations can be performed online in real time. This could allow using the index for control or optimization purposes, taking actions based on minimizing the error between the expected output (i.e., *flow* vector) and the given desired input and, thus, maintaining an optimal *flow* level.

## Ethics statement

This study was carried out in accordance with the recommendations of “the medical ethics committee of Fundación CARTIF” with written informed consent from all subjects. All subjects gave written informed consent in accordance with the Declaration of Helsinki. The protocol was approved by “the medical ethics committee of Fundación CARTIF.”

## Author contributions

CR, DL, JF, and JP conceived and designed the experiments. CR developed the technology for the methods to build the experimental setup, robotics and VR methods, metrics, algorithms, and data processing. KK contributed to the design and layout of the article, tables, additions to the bibliography, and extensive revisions. DL, JF, and JP helped to obtain the funding for the project that financed this research. VG helped with the formulation of the null hypothesis and data processing for the statistics.

## Funding

This research was funded by the Spanish Ministry of Sciences and Innovation (DPI2009-10658) and a research grant (SAN/126/2009) from the Castilla y Leon Council of Health.

### Conflict of interest statement

The authors declare that the research was conducted in the absence of any commercial or financial relationships that could be construed as a potential conflict of interest.

## Footnotes

^1^http://www.biopac.com/data-acquisition-system-mp150-system-glp-win.

^2^http://www.biopac.com/ecg-electrocardiogram-amplifier.

^3^http://www.biopac.com/gsr-eda-galvanic-skin-response-amplifier-electrodermalactivity.

^4^http://www.biopac.com/skin-temperature-amplifier.
