# Supplementary material for: Improving Challenge/Skill Ratio in a Multimodal Interface by Simultaneously Adapting Game Difficulty and Haptic Assistance through Psychophysiological and Performance Feedback
Source: Front Neurosci. 2017 May 1;11:242. doi: 10.3389/fnins.2017.00242 (PMC5410602; doi:10.3389/fnins.2017.00242)
Supplement: Supplementary file 1 [file DataSheet1.docx]

Appendix A

Improving Challenge/Skill Ratio in a Multimodal Interface by Simultaneously Adapting Game Difficulty and Haptic Assistance through Psychophysiological and Performance Feedback.

Carlos Rodriguez-Guerrero^1*^, Kristel Knaepen^2,3^, Juan Carlos Fraile-Marinero^4^, Javier Perez-Turiel^4^, Valentin Gonzalez-de-Garibay^5^, Dirk Lefeber^1^

^1^Robotics and Multibody Mechanics, Vrije Universiteit Brussel, Flanders Make, Belgium.

^2^Institute for Movement and Neurosciences, German Sport University Cologne, Germany.

^3^Human Physiology Research Group, Vrije Universiteit Brussel, Belgium.

^4^Fundacion CARTIF, Biomedical Engineering, Boecillo, Valladolid, Spain.

^5^Department of Statistics and Operative Research, Universidad de Valladolid, Spain.

***Corresponding author**: [carodrig@vub.ac.be](mailto:carodrig@vub.ac.be)

# Supplementary Data

## Fuzzy Rules

- If (HR is High) and (SCR is High) and (SCL is High) then (Assistance is High)(DifficultyInc is HighNegative).
- If (HR is Low) and (SCR is High) and (SCL is Low) and (Performance is Positive) then (Assistance is Low)(DifficultyInc is HighPositive).
- If (HR is Medium) and (SCL is Medium) and (Performance is Neutral) then (Assistance is Low)(DifficultyInc is Positive).
- If (SCR is High) and (Performance is Negative) then (DifficultyInc is HighNegative).
- If (SCR is High) and (Performance is Negative) then (DifficultyInc is None).
- If (SCR is High) and (Performance is Positive) then (DifficultyInc is None).
- If (Performance is Positive) then (DifficultyInc is HighPositive).
- If (Performance is Negative) then (DifficultyInc is HighNegative).
- If (Performance is Neutral) then (DifficultyInc is None).
- If (SCR is High) and (Performance is Positive) then (DifficultyInc is HighPositive).
- If (HR is Low) and (SCL is Low) and (Performance is Neutral) then (Assistance is Low).( DifficultyInc is HighPositive).
- If (HR is Low) and (Performance is Neutral) then (Assistance is Low)( DifficultyInc is HighPositive).
- If (SCL is Low) and (Performance is Neutral) then (Assistance is Low)( DificultyInc is HighPositive).
- If (HR is High) and (Performance is Negative) then (DifficultyInc is HighNegative).
- If (SCL is High) and (Performance is Negative) then (DifficultyInc is HighNegative).
- If (HR is Low) and (Performance is Negative) then (DifficultyInc is HighPositive).
- If (SCL is Low) and (Performance is Negative) then (DifficultyInc is HighPositive).
- If (HR is High) then (DifficultyInc is HighNegative).
- If (SCL is High) then (DifficultyInc is HighNegative).

# Supplementary Figures and Tables

To better understand the obtained results, the SAM scale is provided as a visual aid.


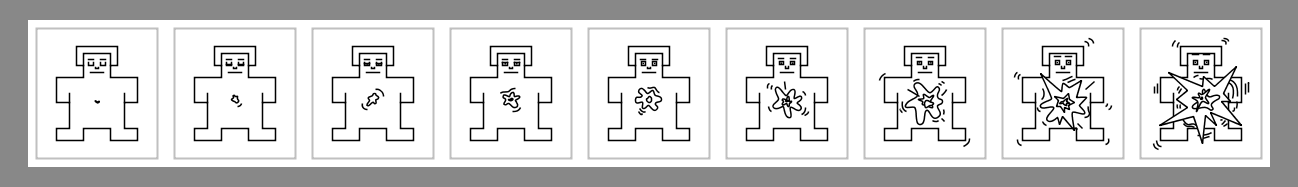

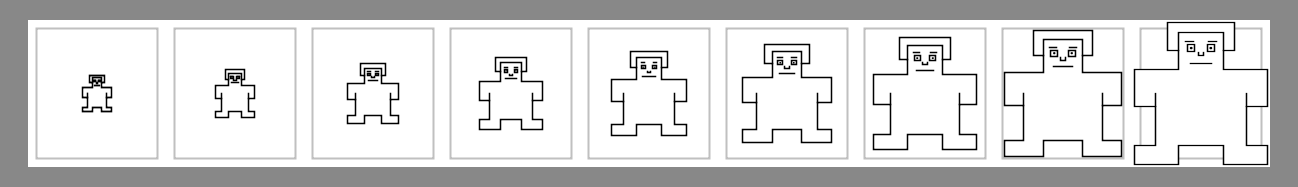

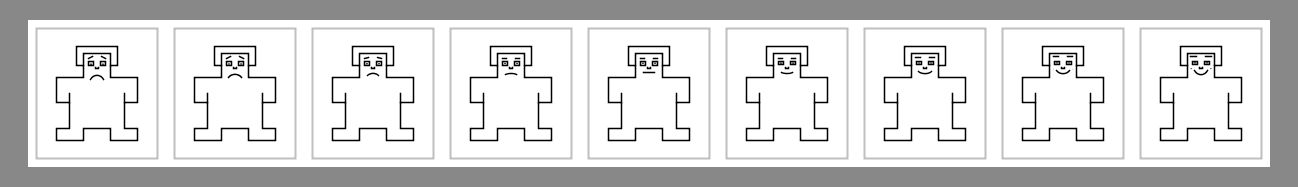


**1 2 3 4 5 6 7 8 9**

**Supplementary Figure 1.** **SAM scale**. From top to bottom: Arousal, Dominance, Valence. The scale ranges from 1 to 9.
